# Isolated Multiple Perforations in the Descending Colon of a Term Baby Who Presented With Vomiting: A Case Report and Literature Review

**DOI:** 10.7759/cureus.109917

**Published:** 2026-05-30

**Authors:** Ahmed J Buali, Rashed Almusalam, Fahad A Al-Qashar, Abdulrahman Alshafei, Eman Shajira

**Affiliations:** 1 Pediatrics, Bahrain Defence Force Hospital, Riffa, BHR; 2 General Surgery, Bahrain Defence Force Hospital, Riffa, BHR; 3 Pediatrics and Neonatology, Bahrain Defence Force Hospital, Riffa, BHR; 4 Pediatric Surgery, Royal College of Surgeons in Ireland (RCSI), Dublin, IRL; 5 Pediatrics, Military Hospital, Royal Medical Services, Riffa, BHR

**Keywords:** case report, descending colon, idiopathic, perforations, sepsis, term newborn

## Abstract

Neonatal intestinal perforation is a life-threatening surgical emergency that is more commonly described in preterm or low-birth-weight infants. Isolated descending colonic perforation in a term neonate is rare and may be difficult to recognize when symptoms are non-specific.

We report a male newborn delivered at 37+2 weeks, weighing 2.6 kg, with Apgar scores of 9 and 9 at 1 and 5 minutes. Breastfeeding was started at two hours and was initially tolerated. At 12 hours, he developed poor feeding and brownish vomiting, while stool passage was present from day one. Initial laboratory tests and chest and abdominal X-ray findings were not diagnostic. He later developed persistent bilious nasogastric aspirate, rising inflammatory markers, thrombocytopenia, *Escherichia coli* bacteremia, and respiratory deterioration. A contrast study did not show malrotation or intestinal obstruction. In view of further clinical deterioration and abdominal distension, an abdominal X-ray was repeated and demonstrated pneumoperitoneum. Emergency laparotomy revealed fecal peritonitis and multiple isolated left descending colon perforations. Histopathology showed a necrotic, perforated descending colon and excluded Hirschsprung disease. After Hirschsprung disease, necrotizing enterocolitis, and intestinal obstruction were considered less likely, idiopathic colonic perforation was considered the most likely explanation. Later, severe sepsis may have aggravated the bowel injury rather than caused the initial perforation. This case emphasizes the importance of repeated clinical assessment and imaging, and early surgical reassessment in deteriorating neonates.

## Introduction

Vomiting in newborns may be the only presenting sign of life-threatening conditions, such as intestinal obstruction, and therefore warrants prompt evaluation [1]. Bilious vomiting is particularly concerning because it may indicate a surgical emergency, and early investigation is important to guide appropriate management [2].

Neonatal intestinal perforation is a life-threatening condition that requires early diagnosis, prompt surgical consultation, and timely management to improve outcomes [3]. Spontaneous intestinal perforation has a reported mortality rate of approximately 20-40% [3]. Intestinal perforation most commonly involves the terminal ileum and colon, while descending colonic perforation is rare [3,4]. It is more commonly reported in very-low-birth-weight infants, especially those weighing less than 1500 g or 1000 g, and is uncommon in term newborns [3].

The causes of neonatal gastrointestinal perforation vary. In one series, non-necrotizing enterocolitis causes were more frequent overall; however, necrotizing enterocolitis was the most common single cause, followed by volvulus, intestinal atresia, intussusception, incarcerated inguinal hernia, and idiopathic perforation [5]. In contrast, among cases of colonic perforation, Hirschsprung disease was the leading cause, followed by idiopathic perforation and necrotizing enterocolitis [6].

Most published reports describe intestinal perforation in preterm or very-low-birth-weight infants, often at more typical sites. We report a rare case of descending colonic perforation in a term newborn who presented with vomiting from the first day of life.

## Case presentation

A male newborn was delivered by spontaneous vaginal delivery at 37+2 weeks of gestation, with a birth weight of 2.6 kg. He was born to a 36-year-old mother with 3 previous uncomplicated vaginal deliveries, and the pregnancy was uneventful. His anthropometric measurements were appropriate for gestational age; his Apgar scores were 9 and 9 at 1 and 5 minutes, respectively; and the initial newborn examination was unremarkable.

**Figure 1:**
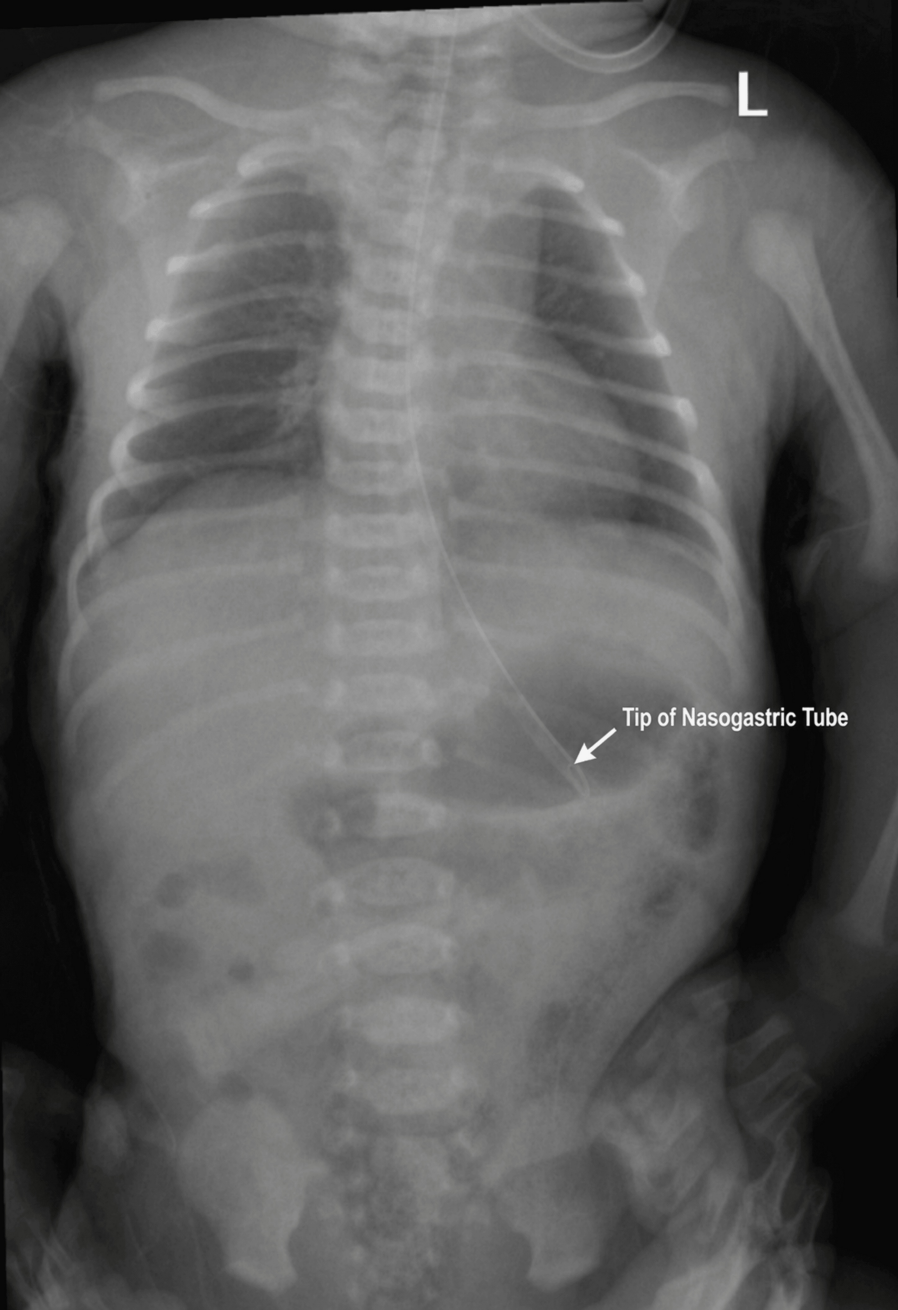
Initial anteroposterior chest and abdominal radiograph The radiograph shows very mild gaseous distension of the stomach and bowel loops. A nasogastric tube is seen, with its tip projecting over the stomach. No obvious pneumoperitoneum is identified.

Breastfeeding was started at two hours of life, and the newborn initially tolerated feeds well. At 12 hours of life, the mother noticed poor feeding, and the newborn developed brownish vomiting. The exact feeding volume was not measured because the newborn was directly breastfed. He passed meconium on day 1. A sepsis work-up was initiated, and chest and abdominal radiographs were performed (Figure 1).

The newborn was started on IV ampicillin 100 mg/kg/day and IV gentamicin 4 mg/kg/day and was kept nil per os (NPO) as per hospital guidelines. Initial laboratory findings did not suggest sepsis, with a WBC count of 7.82 × 10⁹/L, hemoglobin of 19.2 g/dL, platelet count of 172 × 10⁹/L, and CRP of 0.98 mg/L (Table 1).

**Table 1:** Serial laboratory findings during clinical deterioration

Time point	WBC, x10⁹/L	Hemoglobin, g/dL	Platelets, x10⁹/L	CRP, mg/L	Blood culture	Interpretation
Initial sepsis workup	7.82	19.2	172	0.98	Pending	Initial values not strongly suggestive of severe infection
Follow-up labs after 48h of initial sepsis workup	2.64	15.4	131	81.4	Negative	Rising inflammatory markers with leukopenia and thrombocytopenia
Repeated labs with repeated sepsis workup	1	14	78	99.3	Positive for gram-negative rods, later identified as E. coli	Severe neonatal sepsis
48h after the second sepsis workup	5.95	12.8	62	126	Subsequent cultures negative	Ongoing inflammation with thrombocytopenia
Reference range	6 ~ 21	10 ~ 14	150 ~ 450	0 ~ 5	Negative	-

As he remained clinically stable and the initial investigations were reassuring, feeds were restarted. He later developed jaundice requiring phototherapy. Follow-up laboratory investigations after 48 hours showed a WBC count of 2.64 × 10⁹/L, hemoglobin of 15.4 g/dL, platelet count of 131 × 10⁹/L, and CRP of 81.4 mg/L. A repeat chest and abdominal X-ray was unremarkable.

**Figure 2:**
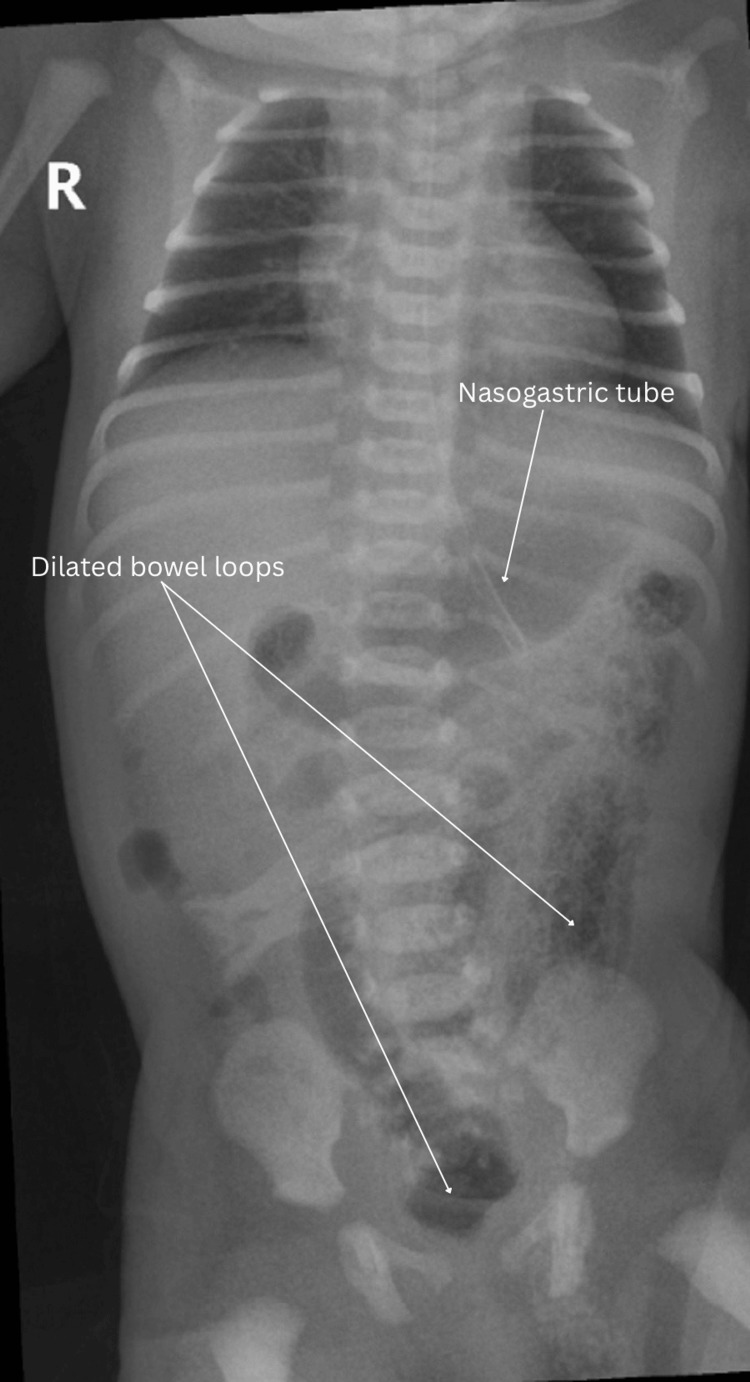
Anteroposterior chest and abdominal radiograph showing mildly dilated bowel loop, nonspecific in appearance, with preserved distal bowel gas.

In view of the markedly rising CRP, leukopenia, and thrombocytopenia, a second sepsis workup was initiated, and CBC, CRP, and chest X-ray were repeated. IV meropenem 100 mg/kg/day was added, and the ampicillin dose was doubled. Follow-up CBC showed a WBC count of 1 × 10⁹/L, hemoglobin of 14 g/dL, platelet count of 78 × 10⁹/L, and CRP of 99.3 mg/L, which were concerning for sepsis. Chest and abdominal X-ray showed mildly dilated bowel loops, and bilious aspirate continued to drain from the nasogastric tube. Therefore, the pediatric surgery team was consulted because of persistent bilious aspirate, abnormal abdominal imaging, and worsening laboratory markers (Figure 2).

**Figure 3:**
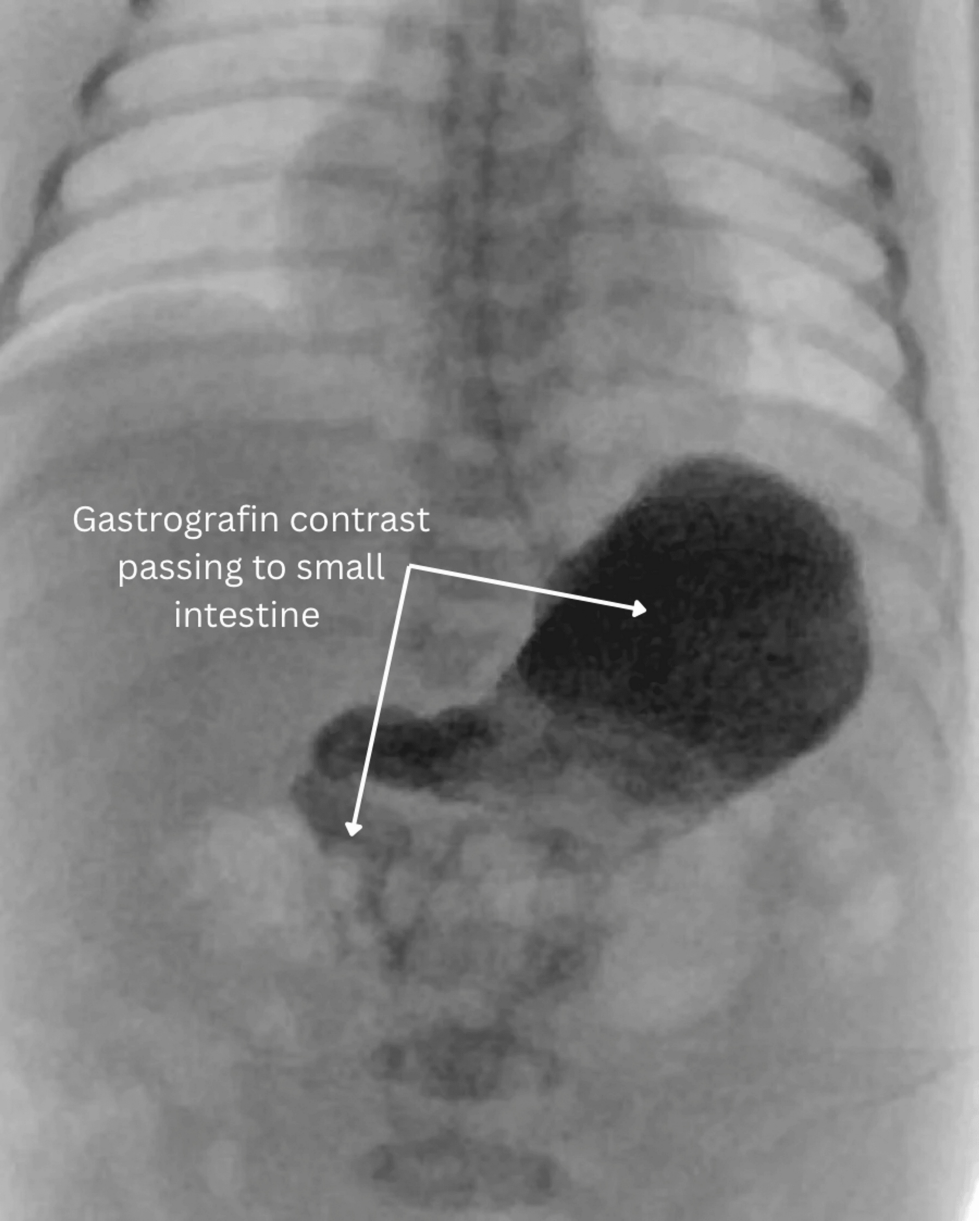
Upper gastrointestinal contrast study Gastrografin contrast is seen filling the stomach and passing into the proximal small bowel. No malrotation, volvulus, obstruction, or delayed passage of contrast is seen in this study.

An urgent Gastrografin contrast study was performed to rule out malrotation or intestinal obstruction, and it did not show either abnormality. Despite this reassuring finding, the newborn continued to have bilious nasogastric aspirate, raising concern for evolving intra-abdominal surgical pathology (Figure 3).

**Figure 4:**
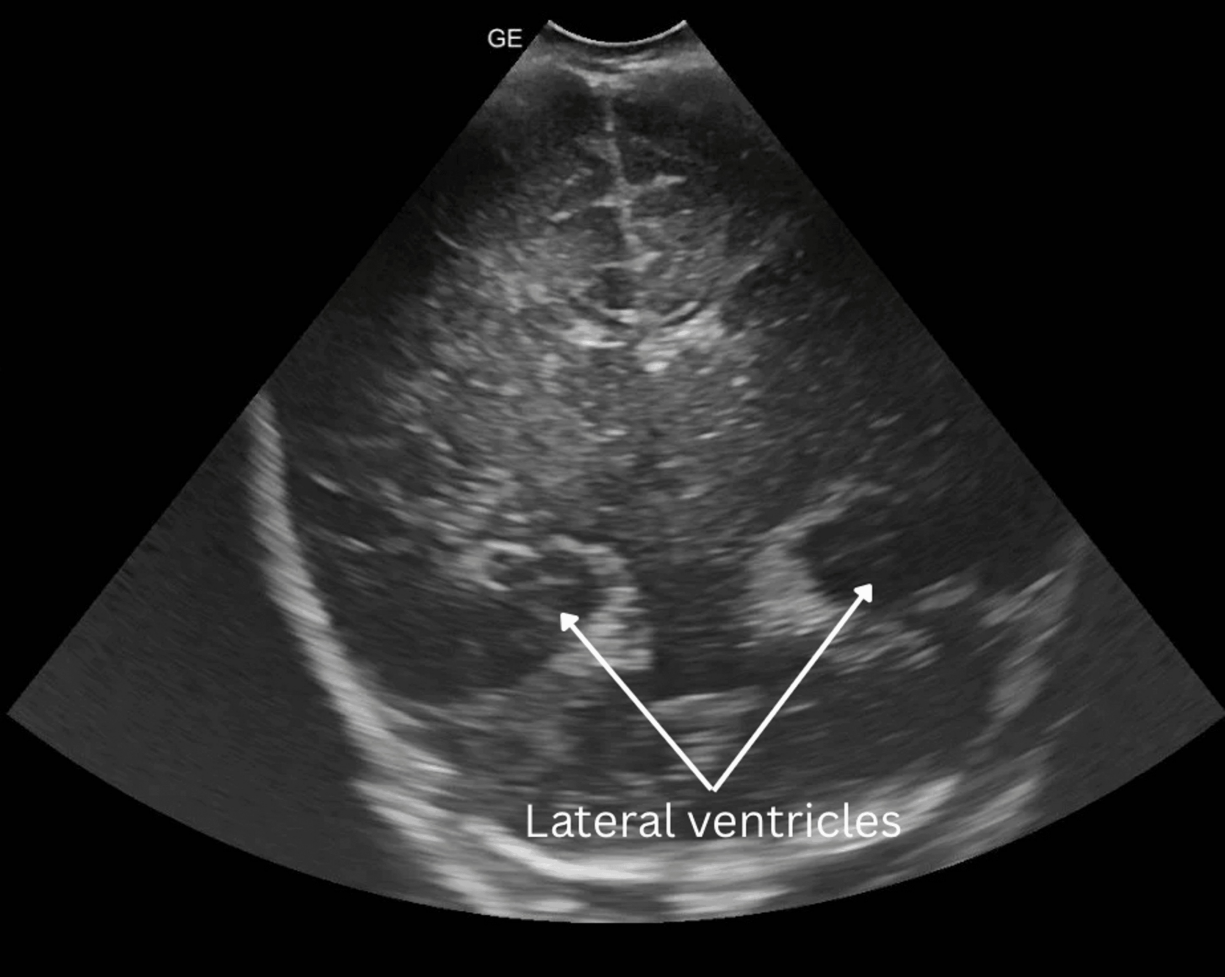
Brain ultrasound Brain ultrasound showed no obvious sonographic abnormality, with no evidence of intraventricular hemorrhage or ventriculomegaly.

Repeat blood culture was positive for Gram-negative rods, later identified as *Escherichia (E.) coli*. Subsequent blood cultures were negative. Lumbar puncture was performed to rule out meningitis, and cerebrospinal fluid culture and the meningitis panel were negative. The newborn received one platelet transfusion before lumbar puncture due to thrombocytopenia. Brain ultrasound showed no obvious sonographic abnormality (Figure 4).

Later that day, he developed respiratory distress with grunting and shallow breathing and was intubated due to clinical deterioration. The newborn showed further clinical deterioration with persistent bilious nasogastric aspirate. Repeat laboratory investigations showed a WBC count of 5.95 × 10⁹/L, hemoglobin of 12.8 g/dL, platelet count of 62 × 10⁹/L, and CRP of 126 mg/L. These findings were consistent with ongoing sepsis and worsening thrombocytopenia, requiring additional platelet transfusion support.

**Figure 5:**
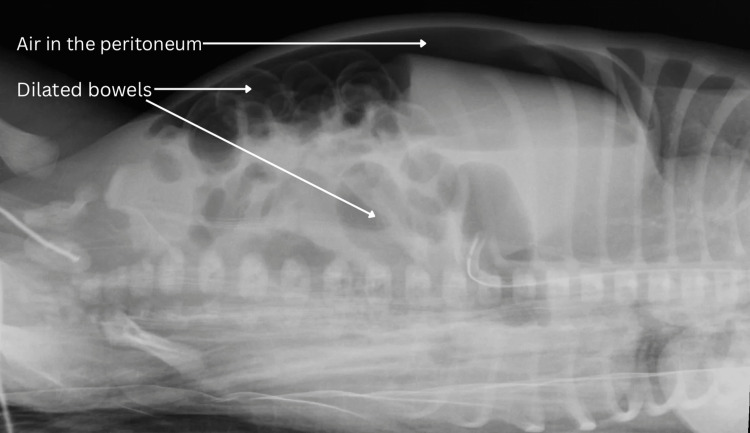
Repeated abdominal radiographs showing pneumoperitoneum Repeat radiographs demonstrated free intraperitoneal air, best seen on the lateral decubitus, lateral view, consistent with pneumoperitoneum. Multiple dilated bowel loops are also seen. These findings were concerning for bowel perforation and prompted urgent surgical intervention.

The following day, his abdomen became markedly distended and tense, with a 2 cm increase in abdominal girth. Therefore, an abdominal X-ray was repeated and showed new pneumoperitoneum (Figure 5).

The radiological findings raised strong concern for intestinal perforation. Therefore, the pediatric surgery team was reconsulted, and the newborn was prepared for urgent surgery. IV metronidazole was added for anaerobic coverage, with a loading dose of 15 mg/kg followed by 7.5 mg/kg every eight hours.

Emergency laparotomy was performed. Intraoperative findings showed fecal peritonitis, confirming bowel perforation. Multiple isolated perforations were identified in the left descending colon. The affected segment of the descending colon was resected, while the transverse colon and rectosigmoid segment were preserved. The remaining transverse colon was matured as a colostomy, and the sigmoid colon was fixed to the fascia and matured as a mucous fistula. Multiple fibrin plaques and inter-bowel adhesions were noted. The cecum was slightly high with a wide mesentery; however, no Ladd bands were identified to suggest malrotation with obstruction. A rectal biopsy was taken to rule out Hirschsprung disease and other pathologies. 

Rectal biopsy showed normal glandular architecture, and ganglion cells were present, ruling out Hirschsprung disease. Histopathological examination of the resected descending colon showed mucosal ulceration, hemorrhage, extensive necrosis, and perforation; however, no granulomas, infectious organisms, dysplasia, or malignancy were seen. No definite vascular thrombosis or vasculitic changes were reported in the available histopathological examination; however, the vascular component was not separately described in the pathology report. Overall, the findings were consistent with a necrotic, perforated descending colon without evidence of aganglionosis, while focal ischemic injury could not be fully excluded.

After surgery, the newborn was continued on meropenem, gentamicin, and metronidazole. The diagnosis of intestinal perforation was supported by clinical deterioration, pneumoperitoneum, operative findings, and histopathology. Overall, the final diagnosis was multiple descending colonic perforations with necrosis and fecal peritonitis of unknown etiology. Histopathology excluded Hirschsprung disease as the underlying cause.

Postoperatively, the newborn showed good clinical progress. At the most recent follow-up, he was gaining weight and tolerating full feeds. The stoma was healthy and active, with no reported complications. The family received stoma care education. The patient continued under surgical follow-up, with colostomy closure planned in the future.

## Discussion

This case represents a rare presentation of descending colonic perforation with necrosis and fecal peritonitis in a term neonate. The newborn initially presented with poor feeding and non-bilious vomiting, followed by persistent bilious nasogastric aspirate. The clinical course progressed rapidly, with subsequent abdominal distension and pneumoperitoneum, which required urgent laparotomy. The main clinical lesson from this case is that the initial presentation can be non-specific, and early imaging findings may not be diagnostic, which can make early recognition difficult. The presence of intestinal perforation was confirmed by pneumoperitoneum, intraoperative findings, and histopathology; however, the exact etiology remained uncertain.

Intestinal perforation is a life-threatening surgical emergency that can lead to serious complications if not treated promptly. In neonates, intestinal perforations most commonly involve the terminal ileum or proximal colon. In contrast, isolated perforation of the descending colon is extremely uncommon in term neonates, making this case a rare presentation [3,4,6]. Only a limited number of similar neonatal cases have been reported in the literature [7]. This case is important because of its unusual clinical course, which included early gastrointestinal symptoms, worsening abdominal signs, later evidence of sepsis, and delayed radiological findings.

This case was challenging because the initial presentation was misleading, with vomiting and poor feeding, which are common neonatal complaints. In addition, the newborn initially had a soft, normal abdomen, and stool was passed from day 1. These findings were reassuring. However, passage of meconium or stool during the first day of life does not completely exclude Hirschsprung disease or evolving intestinal obstruction, particularly in early neonatal presentations [8,9].

In our case, the progression from brownish vomiting to persistent bilious nasogastric aspirate prompted radiological evaluation to rule out obstruction or malrotation. Dilated bowel loops on abdominal X-ray raised concern for intestinal obstruction. Therefore, a contrast study was performed to rule out common causes of obstruction such as volvulus, malrotation, and intestinal atresia [10]. Although the contrast study did not demonstrate malrotation or obstruction, a normal study does not completely exclude evolving intestinal ischemia or subsequent perforation in clinically deteriorating neonates [2,10-12].

The following day, the newborn developed abdominal distension, and the abdominal X-ray was repeated. It showed pneumoperitoneum, indicating intestinal perforation, and this finding was the decisive turning point for urgent surgical intervention [3,13].

Intraoperatively, multiple perforations were found in the descending colon with fecal peritonitis. Similar reports have described neonatal colonic perforation with fecal peritonitis, although the location of perforation may vary [4]. The affected descending colon was resected, while the rectosigmoid segment, transverse colon, and small bowel were preserved. Preservation of the remaining colon and small bowel was important to minimize the risk of intestinal failure and short bowel syndrome [14,15]. Formation of a colostomy with a mucous fistula allowed diversion of the fecal stream while preserving the distal bowel for future restoration of intestinal continuity [16].

No clear etiology was identified for the descending colonic perforations in this case. Previous series have reported neonatal colonic perforation mainly in association with Hirschsprung disease, necrotizing enterocolitis, intestinal obstruction, or idiopathic perforation [5,6]. In our case, a rectal biopsy was performed to rule out Hirschsprung disease and other pathological causes of neonatal colonic perforation. This approach is supported by Balcı et al., who recommended biopsy in term neonates with non-necrotizing enterocolitis colonic perforation to detect underlying aganglionosis and allow timely management [17]. Histopathological examination showed ganglion cells, ruling out aganglionosis, as Hirschsprung disease is diagnosed by the absence of ganglion cells in the submucosal plexus [9]. The biopsy also helped exclude other structural or pathological disorders, such as granulomatous disease, dysplasia, and malignancy [9]. No intestinal obstruction was identified on contrast study or during surgery. Necrotizing enterocolitis was also considered but was less likely because the neonate was term, radiological findings were not typical, and histopathology did not show supportive features such as pneumatosis intestinalis, diffuse transmural inflammation, or widespread bowel involvement [5,7]. Overall, the findings were consistent with a necrotic perforated descending colon without evidence of aganglionosis, while necrotizing enterocolitis and intestinal obstruction were considered less likely.

The temporal sequence suggests that an underlying bowel pathology may have preceded the development of sepsis. The newborn developed gastrointestinal symptoms, including brownish vomiting at 12 hours of life, before any laboratory or microbiological evidence of sepsis. The initial WBC count, CRP, and platelet count were also normal at presentation. Therefore, sepsis is less likely to be the primary precipitating cause of perforation. After Hirschsprung disease, intestinal obstruction, and necrotizing enterocolitis were considered less likely, idiopathic colonic perforation was considered the most likely etiology. Subsequent *E. coli *bacteremia, increased CRP, leukopenia, thrombocytopenia, clinical worsening, and histopathological necrosis support sepsis as a possible aggravating factor rather than the initial event. Sepsis may worsen bowel injury through intestinal hypoperfusion, microcirculatory dysfunction, increased intestinal permeability, and mucosal barrier damage [18,19]. 

To summarize the diagnostic reasoning, the main differential diagnoses considered in this case, along with the points supporting and opposing each diagnosis, are presented in Table 2.

**Table 2:** Differential diagnoses considered in a term neonate with descending colonic perforation The conclusions in this table reflect the interpretation of the present case based on clinical, radiological, operative, microbiological, and histopathological findings. *E. coli: Escherichia coli; *NEC: necrotizing enterocolitis

Differential diagnosis	Points for	Points against	Conclusion	References
Mechanical obstruction/ volvulus	Early vomiting and progressive abdominal distension may suggest obstruction.	No volvulus, malrotation, atresia, or obstructing lesion was identified on contrast study, during surgery, or on histopathology	Less likely.	[1,10,11,12]
Hirschsprung disease-associated perforation	Hirschsprung disease is a known cause of neonatal colonic perforation.	Rectal biopsy showed ganglion cells, and the resected colon showed no aganglionosis.	Ruled out by histology.	[4,6,9,17]
Necrotizing enterocolitis	NEC can cause bowel necrosis, perforation, clinical deterioration, and sepsis.	The neonate was term, and perforations were limited to the descending colon. Radiological and histopathological findings did not show typical NEC features such as pneumatosis intestinalis, diffuse transmural inflammation, or widespread bowel involvement	Considered, but less likely.	[5,7]
Idiopathic/spontaneous intestinal perforation	No clear cause was found, and gastrointestinal symptoms started before laboratory or microbiological evidence of sepsis.	Multiple perforations in the descending colon are not typical for spontaneous intestinal perforation.	Most likely explanation, although the site and multiplicity are atypical.	[3,5,6]
Sepsis-related bowel injury	Later, E. coli bacteremia, increased CRP, leukopenia, thrombocytopenia, and clinical deterioration suggest that sepsis contributed to the course.	Gastrointestinal symptoms started before laboratory and microbiological evidence of sepsis.	More likely contributed to the injury rather than caused it initially.	[18,19]
Focal ischemic bowel injury	Histopathology showed mucosal ulceration, hemorrhage, necrosis, and perforation.	No definite vascular occlusion or clear primary ischemic cause was identified.	Possible, not proven.	[19]

## Conclusions

This case highlights a rare presentation of multiple isolated perforations of the descending colon in a term neonate, complicated by severe neonatal sepsis, pneumoperitoneum, and fecal peritonitis. The initial presentation was misleading, as poor feeding and vomiting occurred despite a soft abdomen and passage of stool. It also shows that an evolving surgical condition cannot be fully excluded by a single reassuring contrast study when the patient continues to deteriorate clinically. Repeated clinical examination, repeat imaging, and early pediatric surgical reassessment should be considered when bilious nasogastric aspirate persists, with worsening inflammatory markers, thrombocytopenia, abdominal distension, or respiratory deterioration. Although Hirschsprung disease, necrotizing enterocolitis, and intestinal obstruction were considered, these were less likely based on the histopathological, radiological, contrast study, and surgical findings. The exact etiology of the perforation could not be confirmed. Later, severe sepsis may have aggravated the bowel injury, but idiopathic perforation remains the most likely explanation. This case adds to the limited literature describing isolated descending colonic perforation in term neonates and emphasizes the importance of repeated reassessment in evolving neonatal surgical conditions.
